# 35. PREVENTION OF PSYCHOSIS: AN INDIVIDUAL OR POPULATION APPROACH?

**DOI:** 10.1093/schbul/sby014.146

**Published:** 2018-04-01

**Authors:** Mary Cannon

**Affiliations:** Royal College of Surgeons in Irel

## Abstract

**Overall Abstract:** Psychotic disorders and schizophrenia in particular have a profound impact on patients, their caregivers and society. Mental illness is set to overtake cancer and cardiovascular disease to become the most expensive disorder in terms of direct expenditure and disability-adjusted life years over the next decade. Unfortunately, mental health has lagged behind physical disorders in terms of focus on prevention. It is imperative that prevention is taken more seriously for mental health disorders. In this symposium we present novel data and novel perspectives on risk and protective factors for psychosis from the viewpoint of prevention.

Data will be presented from large population based studies from England, France, Italy, Netherlands, Spain, and Brazil and Denmark Danish These include epidemiological studies of first episode psychosis (FEP) (both single centre and multicentre) and large register- based studies.

Olesya Ajnakina will show that only 4.1% of a sample of young adults with first episode psychosis diagnosed over a two year period had actually been seen previously by the prodromal services. This indicates that this “At risk mental state” approach is not useful for prevention of psychosis at a population level. Hannah Jongsma will present data from the EU-GEI large multicentre study showing an association between greater catchment area-level owner-occupancy and lower incidence of psychosis. She also replicated the well-established finding on increased risk for psychosis among minority groups. These findings show that we need to tackle societal factors rather than remaining focused on an individual level approach. Using register data from Denmark, Kristine Engemann Jensen reports a novel protective factor for psychosis – childhood exposure to green space. This shows the importance of the built environment for mental health – particularly for young people. Finally, Sir Robin Murray gives his particular insights on how we can prevent psychosis using data from three first episode psychosis studies. He shows, for instance, that 24% of psychosis cases could theoretically be prevented by eliminating use of high-potency cannabis use in the population. He argues that psychiatrists and psychologists need to get involved in promoting societal and legislative approaches to reducing known risk factors for psychosis. Our discussant Andreas Meyer-Lindenberg will draw on all these findings, along with his own work on risk factors such as urbanicity, in discussing how we can now move to a new prevention-focused paradigm of research on psychosis.

